# Current Challenges in Efficient Lithium‐Ion Batteries’ Recycling: A Perspective

**DOI:** 10.1002/gch2.202200099

**Published:** 2022-09-08

**Authors:** Xiaolu Yu, Weikang Li, Varun Gupta, Hongpeng Gao, Duc Tran, Shatila Sarwar, Zheng Chen

**Affiliations:** ^1^ Program of Materials Science University of California, San Diego La Jolla CA 92093 USA; ^2^ Department of NanoEngineering University of California, San Diego La Jolla CA 92093 USA; ^3^ Program of Chemical Engineering University of California, San Diego La Jolla CA 92093 USA; ^4^ Sustainable Power and Energy Center University of California, San Diego La Jolla CA 92093 USA

**Keywords:** challenges, electric vehicles, profits, recycling, spent Li‐ion batteries

## Abstract

Li‐ion battery (LIB) recycling has become an urgent need with rapid prospering of the electric vehicle (EV) industry, which has caused a shortage of material resources and led to an increasing amount of retired batteries. However, the global LIB recycling effort is hampered by various factors such as insufficient logistics, regulation, and technology readiness. Here, the challenges associated with LIB recycling and their possible solutions are summarized. Different aspects such as recycling/upcycling techniques, worldwide government policies, and the economic and environmental impacts are discussed, along with some practical suggestions to overcome these challenges for a promising circular economy for LIB materials. Some potential strategies are proposed to convert such challenges into opportunities to maintain the global expansion of the EV and other LIB‐dependent industries.

## Graphical abstract

Rapid prospering of the electric vehicle industries makes Li‐ion batteries (LIBs) recycling an urgent need. However, the global LIB recycling effort is hampered by various challenges, including recycling/upcycling techniques, global government policies, and economic and environmental implications. Specific challenges are summarized along with some practical suggestions to overcome these challenges for a promising circular economy for LIB materials.

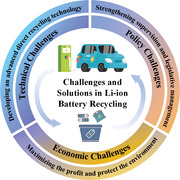

## 1 Introduction

### 1.1 Factors Driving for End‐of‐Life Li‐Ion Battery Disposal

The decarbonization initiatives by governments worldwide, especially in the automotive and energy industries, stimulate demand for various energy storage devices. Li‐ion batteries (LIBs) are dominating the market due to their high energy and power density,^[^ ^1^ ^]^ especially for electronic devices, electric vehicles (EVs), and grid storage systems. As a result, the global market of LIBs is expected to follow a rapid upward trend, projected to reach US$56 billion by 2024.^[^ ^1c^ ^]^ The primary growth will be triggered by the massive EV production, which is expected to reach $253 million by 2030.^[^ ^1c^ ^]^

The rising LIB utilization has increased the demand for critical raw materials such as lithium (Li), nickel (Ni), and cobalt (Co). However, most of these essential materials are regulated by specific countries. More than half of cobalt ore is mined in the Democratic Republic of Congo and refined in China, and about 80% of lithium is controlled by Australia and Chile.^[^ ^2^ ^]^ This uneven distribution of raw materials and production areas has raised concerns about the global supply chain. As a result, lithium and cobalt prices are rising and fluctuating, and in the meantime, the geopolitics could lead to a monopoly of raw material supply by local governments.^[^ ^3^ ^]^ Therefore, from a sustainability perspective, it is essential to establish a secondary supply of critical materials recovered from spent LIBs (from EVs, stationary storage batteries, and home appliances) and from the manufacturing wastes (trimming, end products, off‐spec products, etc.) to mitigate the severity of this potential shortage.

On the other hand, since LIBs can usually be used for 10 years on average,^[^ ^3^, ^4^ ^]^ the amount of spent LIBs is expected to reach more than 5 million tons by 2030.^[^ ^5^ ^]^ The main components of LIBs are cathode materials (LiNi*_x_* Co*_y_* Mn*_z_* O_2_ (0 < *x*, *y*, *z* <1, *x* + *y* + *z* = 1, known as NCM), LiCoO_2_ (LCO), LiFePO_4_ (LFP), etc.), anode materials (graphite), current collectors (aluminum (Al) and copper (Cu)), electrolyte salts such as lithium hexafluorophosphate (LiPF_6_), organic solvents (ethylene carbonate (EC), diethyl carbonate (DEC), ethyl methyl carbonate (EMC), dimethyl carbonate (DMC), etc.). All these different components contain hazardous materials and result in metal, dust, organic, and fluorine contaminations.^[^ ^6^ ^]^ Landfilling or incineration can harm ecosystems. For example, once the electrode materials enter the environment, metal ions from the cathode, carbon dust from the anode, strong alkali, and heavy metal ions from the electrolyte may cause severe environmental pollutions, hazards, etc., including raising the pH value of the soil,^[^ ^7^ ^]^ and producing the toxic gases (HF, HCl, etc.). In addition, the metals and electrolytes in batteries can harm human health. For example, the cobalt may get into the human body via underground water and other channels, causing symptoms such as intestinal disorders, deafness, and myocardial ischemia.^[^ ^8^ ^]^ Although LIBs can enable the efficient use of renewable energy while potentially reducing carbon emissions, poorly managed LIBs’ waste can also negatively impact the economical and social development of a society.^[^ ^3^ ^]^

Therefore, in order to maintain the growth of LIB applications, to protect resources (reduce the resource wastes, such as Li, Ni, Co, and Mn), to reduce the pressure of potential resource shortages, to ease the environmental pressure, and to reduce environmental pollution, there is an urgent need to establish a sustainable and effective recycling system for spent LIBs.

### 1.2 Disposal Pathways for End‐of‐Life LIBs: Repurposing and Recycling

**Figure 1:**
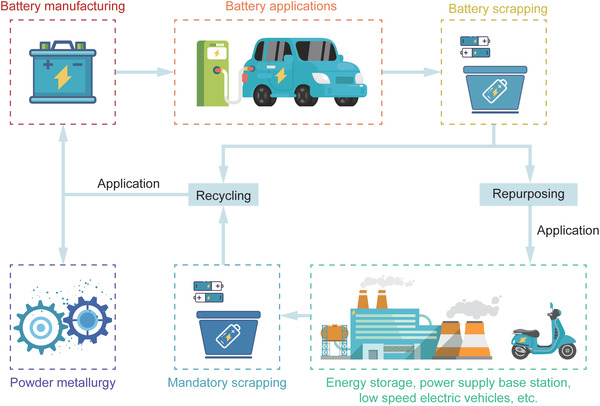
Life cycle of EV batteries via repurposing and recycling.

According to the United States Advanced Battery Consortium (USABC), an EV battery, module, or pack is considered to reach end of life (EoL) when its capacity drops to ≈80% of its original nominal capacity or power.^[^ ^3^ ^]^ Depending on the type, quality, and state of health (SoH) of the EV battery, EoL LIBs from EV have two main options: repurposing and recycling.^[^ ^9^ ^]^ **Figure** **1** summarizes the life cycle of a power cell in both processing modes.

Repurposing (or cascade utilization) of spent EV batteries means that when a battery pack reaches the EoL below 80% of its original nominal capacity,^[^ ^3^, ^9^ ^]^ individual module or cell can be analyzed to reconfigure new packs with specific health and a calibrated battery management system (BMS) so that they can be used in appropriate applications with the same or lower power requirements (grid energy storage, home energy storage, low power electric vehicles, etc.).^[^ ^10^ ^]^ However, currently, there are significant technical and market difficulties in the cascade utilization of spent EV batteries. Technical difficulties include evaluating and testing the SoH of spent batteries, setting technical standards based on different designs since the EV power and energy storage batteries follow different technical standards, and the vital need to address safety issues during the segregation and repurposing process. Additionally, several market difficulties, including complex market processes for secondary batteries flowing into the market, consumer psychological acceptance of reused batteries, the distribution risk, and responsibility for batteries used for other than their intended function, etc., are all obstacles toward the development of a healthy and long‐term market for cascade utilization. Therefore, if a series of breakthroughs in various technologies occur in the future, such as accurate SoH prediction and detection,^[^ ^11^ ^]^ simplified screening, and regrouping spent LIB process with the assistance of machine learning^[^ ^12^ ^]^ are developed, the economic benefit of cascade utilization will gradually become clearer. Furthermore, cascade utilization will allow for the optimal use of the energy value of spent batteries within this SoH range.

On the other hand, once the batteries are reduced to less than 80% of their nominal capacity or no longer suitable for repurposing, they should be recycled. The established processes of recycling the spent batteries are relatively simple and are currently the primary method for safe EoL battery disposal. The recycling process for EoL power batteries mainly involves industrial processes such as pre‐discharging, disassembling, sorting, shredding, purifying, and re‐manufacturing. The cathode material accounts for the most significant proportion (40%) of the total battery value since it contains a large number of precious metal elements (Li, Co, and Ni), and the recycling research mainly focuses on cathode recycling for the profit purpose.^[^ ^3^ ^]^ By reducing the amount of Co in cathode materials, such as Ni‐rich NCM, cathode materials are evolving with enhanced energy density and reduced material costs.^[^ ^13^ ^]^ Therefore, the profit margin by simple metal extraction will become thinner, and the related battery recycling business model needs a significant upgrade.^[^ ^13b,c^ ^]^ The recycling of anodes, electrolytes, and current collectors must be considered to increase profits. Compared with cascade utilization, recycling with traditional technology for spent LIBs is relatively mature but still faces many challenges regarding technology, profitability, logistics, etc.^[^ ^3^, ^14^ ^]^

In the following section, we discuss LIB recycling trends from three perspectives: 1) developing advanced LIB recycling technology by direct recycling technology, strengthening supervision and legislative management, maximizing the benefits and protecting the environment; 2) detailed analysis of difficulties and opportunities encountered during the development of LIB recycling from technical, political, economic, and environmental perspectives; and 3) promising strategies that may be used to address current challenges and accelerate the emerging LIB recycling industry development.

## 2 Global Challenges and Opportunities Ahead in the LIB Recycling

### 2.1 Developing an Advanced Direct Recycling Technology for LIBs

**Figure 2:**
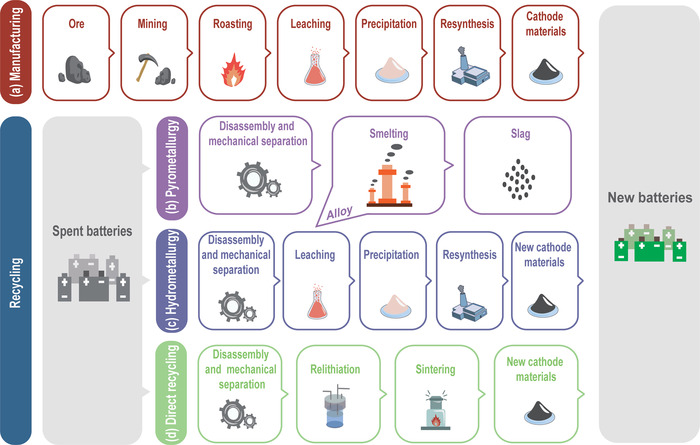
Process workflows for primary cathode manufacturing, and recycled cathode manufacturing including pyrometallurgical process, hydrometallurgical process, and direct recycling methods.

As shown in **Figure** **2**, there are three main methods for recycling: pyrometallurgical, hydrometallurgical, and direct recycling processes. Among them, the first two are already commercialized for industrial production, while the latter is still at the laboratory level, and efforts are underway to expand production for more real‐world data.^[^ ^15^ ^]^

#### 2.1.1 Challenges in Pyrometallurgy and Hydrometallurgy Methods

**Table** **1** summarizes the advantages and disadvantages of the three recycling processes. The pyrometallurgy method is widely adopted in Europe, the United States (US), and Japan for spent LIB recycling,^[^ ^15^ ^]^ as it has been well established for commercialization which can massively and efficiently produce mixed metal slags. Moreover, the pyrometallurgical method is universally compatible with all types of batteries.^[^ ^16^ ^]^ As shown in Figure 2, the organic materials (plastics, separators, electrolytes, etc.) and aluminum in the battery are oxidized by high‐temperature smelting, the transition metal oxides get reduced to transition metals, and form a mixed metal alloy.^[^ ^13c^ ^]^ Thus, the metallic elements in the electrode and package materials are converted to stable metal oxides. However, some valuable elements such as Li and Al remain in the slag, making it difficult to recover and reuse them.^[^ ^17^ ^]^ In addition, this recycling process is prone to generate large amount of harmful emissions (fluorine compounds, toxic organic compounds, and some greenhouse gasses). Additional costs are required for exhaust gas treatment, and additional electricity consumption is needed for melting. Therefore, based on the Everbatt model, pyrometallurgy requires more costs than both of the hydrometallurgical method and the direct recycling method to recycle 1 kg of spent LIBs containing LCO or NCM cathodes.^[^ ^18^ ^]^

**Table 1:** Comparison of hydrometallurgical, pyrometallurgical, and direct recycling processes

Type	Pyrometallurgical process		Hydrometallurgical process		Direct recycling process
Advantages	Flexible input materials No presorting Short process flow Commercially available Could replace mining and metallurgy to a certain extent		High recovery rate and high purity of valuable metals (including Li) Lower energy consumption Products could be used for cathode production Commercially available		Low cost, low consumption Less pollution More profitable Embedded energy inside of material structure maintained Products can directly be used as cathode materials
Disadvantages	Li and Al remaining in slag Embedded energy inside of material structure loss Extra cost for treating gas pollution Not targeted recycling and need metal separations High energy inputs		Presorting and pretreatment required Embedded energy inside of material structure loss Lengthy and complex process Pollutions (greenhouse gases, wastewater, etc.) caused by extensive usage of chemicals		Presorting required with more separation processes More requirements for input materials (single material recovery, degradation conditions, etc.) Still at lab scale

The hydrometallurgical process is the predominant recovery method in China.^[^ ^15^ ^]^ As shown in Figure 2, the hydrometallurgical process involves acid or alkali leaching followed by lithium extraction, separation, and conversion to recover a variety of metal compounds. The recovered compounds can be used to synthesize precursors of new cathode materials. This method allows complete recovery at lower temperatures and flexible disposal with high metal recovery efficiency. To recycle 1 kg of spent LIBs, while hydrometallurgy requires lower energy inputs (i.e., diesel, natural gas, or electricity consumption) and overall recycling cost is lower than pyrometallurgy, it requires more than two times materials input (reagents such as sulfuric acid, sodium hydroxide, and hydrogen peroxide) than pyrometallurgy.^[^ ^19^ ^]^ In addition, fluorine compounds and low‐molecular‐weight organic compounds tend to be mixed into the solution during the treatment process, making post wastewater treatment relatively expensive and complicated.^[^ ^20^ ^]^

The above two existing recycling methods commonly used in industry are based on the destruction of the cathode structure and the extraction of valuable elements (Li, Ni, Co, and Mn). The electrode materials usually have stable structures. They often require extreme conditions to destroy the chemical bonds of the cathode materials, such as high temperature and strong acid methods, along with lengthy subsequent extraction process, high energy consumption, and high cost. Moreover, as cathode materials evolve into Ni‐rich compositions, profit margins decline with solely metal extraction and many valuable parts of LIBs are lost in the recycling process.^[^ ^13b^ ^]^ Therefore, new approaches like direct recycling or even upcycling, rather than traditional pyrometallurgical and hydrometallurgical processes, are under development to upgrade the recycling business.

#### 2.1.2 Challenges and Opportunities in Direct LIB Recycling

The direct recycling method has been emerging in recent years for LIB recycling. During this recycling process, the spent cathode material from LIBs can be treated electrochemically or physicochemically to restore the damaged structure and electrochemical properties so that they can be utilized again directly as new cathode materials or as precursors for the preparation of new electrodes with a significant amount of embedded energy retained within the cathode particle structures.^[^ ^21^ ^]^

The research publications based on the direct recycling process are continued a significant growth throughout the recent years.^[^ ^20b^ ^]^ However, many issues and challenges must be resolved before direct recycling evolves from lab scale into commercialization and rapid development.

*The Development of Advanced Direct Recycling Process*: Direct recycling for spent LIB cathode materials based on different chemistries has been demonstrated recently.^[^ ^22^ ^]^ Though showing great potential for commercialization, the detailed process flow via direct recycling requires more effort before scaling to the market level.^[^ ^23^ ^]^ Technically, the ultimate goal of direct recycling is to establish a closed‐loop recycling method^[^ ^24^ ^]^ for large‐scale processing of spent LIB cathodes under mild temperature and pressure. Chen and co‐workers^[^ ^4^, ^25^ ^]^ developed low‐temperature hydrothermal relithiation methods for recycling the spent LFP and NCM cathodes under temperature below 100 °C and pressure less than 1 bar with assistance of reducing agents. In these processes, reducing agents assist to reduce the activation barrier of the relithiation process. Thus, the Fe^3+^ in degraded LFP and Ni^3+^ in degraded NCM can be reduced for stoichiometric Li accommodation. This recycling method represents a new trend toward a closed‐loop environment‐friendly recycling approach without additional safety concerns.

In addition, recycling can be upgraded to upcycling, which can change the chemistry of recycled cathode materials based on the market demand. For instance, low nickel NCM111 can be upcycled to NCM 622,^[^ ^26^ ^]^ so that the energy density can be improved to fit the state‐of‐the‐art market demands. Another benefit of upcycling is converting polycrystals to single crystal.^[^ ^25^ ^]^ This upcycling can be accomplished by a solid‐state sintering process, or a universal acid etching method,^[^ ^27^ ^]^ resulting in better performance in terms of cycle life and rate performance. Furthermore, cathode surface modification or doping for enhanced cycling performance can also be embedded into the upcycling category.^[^ ^28^ ^]^ For instance, spent LCO can be relithiated and doped with Mg to Mg‐recovery LCO so that their cycling stability at high voltage can be improved and even exceed that of new cathode materials.^[^ ^29^ ^]^

*Excessive Dependency on Manual Labor with Safety Concerns*: One of the major challenges with direct recycling methods is that the highly repetitive procedures, especially mechanical disassembly, which relies heavily on manual labor.^[^ ^30^ ^]^ Extensive manual labor results in inefficiencies, high labor costs, and working environment safety risks. LIB cells can be cylindrical‐, prismatic‐, or pouch‐shaped, and since cells are assembled into modules and then into packs, variations and uncertainties in composition and shape create significant challenges for recycling. In addition, each manufacturer does not share a unique design or recipe.^[^ ^17^ ^]^ Recycling would be more efficient if all packs and modules were standardized with appropriate labels that can be identified externally since automatic recognition and disassembly would be extensively implemented. Besides, the full range of information, including the identification of manufacturer, manufacturing date, and type of battery, significantly improves handling safety and disassembly efficiency.^[^ ^17^ ^]^ There are also risks associated with the battery disassembly process, whether manual or mechanical, as it requires an external force to complete the disassembly process. Relevant safety arrangements and regulations need to be in place to reduce the risk of battery disassembly since flammable materials such as electrolytes or accidental short circuits between cathode and anode direct contacts in the battery may result in explosion and fire hazards.

As manual disassembly of LIBs is inefficient and labor‐intensive, it is essential to develop automated disassembly based on the standard size and shape of battery packs to reduce costs and labor. Therefore, using many of the fourth industrial revolution technologies,^[^ ^31^ ^]^ automation can be achieved through artificial intelligence, automated or semiautomated machine learning, and deep learning methods for rapid disassembly and efficient recycling of retired LIBs, as well as multivariate data‐driven methods, to estimate LIB lifespan accurately.^[^ ^11^ ^]^ Based on this, a robot was proposed for safe and rapid battery retrieval,^[^ ^32^ ^]^ remaining battery quantity detection, and secondary use of retired batteries. In an actual case study of a battery pack disassembly experiment, the robotic disassembly system was found to reduce the processing time by 80–90% compared to a manual disassembly system.^[^ ^32^ ^]^ Therefore, it is necessary to realize an automatic dismantling process, residual energy detection, secondary utilization, and chemical recovery to promote the development of LIB recycling.

*Meeting the Requirements of Industrial Materials*: For direct recovery, existing laboratory scale electrode tests (coin cell, electrode load ≈1–2 mAh cm^−2^, and active material composition ≈80–90 wt%) lag far behind the general industry requirements (electrode load ≈3 mAh cm^−2^ and active material composition ≈95 wt% multilayer pouch cells), thus makes it more challenging to convince the production lines to accept recycled materials.^[^ ^3^, ^33^ ^]^ More reliable tests, such as multilayer pouch cells, are needed to compare side by side with virgin materials.

However, the difficulty of obtaining large quantities of spent cathode material from a single laboratory may prevent further expansion of the direct recycling process. Therefore, universities, laboratories, and industries are encouraged to work together and cooperate. For example, the industry should provide a stable supply of spent batteries and to point out the natural technical barriers that exist in industrial production; universities and laboratories based on first‐hand technical issues can carry out research and try to expand the scale of experiments. In this way, industry, universities, and laboratories will conduct lab‐scale and industrial‐level tests simultaneously on recycled materials, break through technical barriers, and jointly solve the possibility of large‐scale recycling of LIBs in practical production lines. With cooperation and intensive research, large‐scale direct recycling employing in the industry would minimize the pollution and maximize the benefits.

As described above, significant innovation in integrating existing recycling methods for LIBs is highly desired to achieve high recycling rates at a cost competitive of buying virgin materials and battery suppliers.

### 2.2 Strengthening Supervision and Legislative Management for LIB Recycling

In addition to pushing advanced technology development, government regulations and legislative management to support LIB recycling must be implemented globally. As most countries are still at a very early stage of recycling LIB industry development, the state‐of‐the‐art collection and recycling amount of spent LIBs are not as well established as expected.^[^ ^34^ ^]^ Two of the major reasons are the inadequate closed‐loop spent LIB recycling channels and the lack of market regulations for the LIB recycling industry.

China, the US, and European Union (EU) ranked top three of the world's largest LIB markets have invested heavily in novel LIB recycling technologies and documented various relevant regulations.^[^ ^35^ ^]^ The Extended Producer Responsibility (EPR) regulation,^[^ ^36^ ^]^ which applies to Asian countries, North America, and the EU, requires producers (manufacturers, automakers, and retailers) to collect and recycle spent LIBs from the vehicles and equipment sold to consumers.^[^ ^37^ ^]^ Governmental recycling centers are national‐level recycling centers set up by local governments following relevant national laws to promote proper regulations and management of the LIB recycling market, improve recycling networks, and increase recycling amount through regulated channels.^[^ ^38^ ^]^ Currently, most countries do not have government‐affiliated LIB recycling centers, but it is expected to be built to align with national realities for future development.^[^ ^39^ ^]^

#### 2.2.1 China

In China, national and local governments have enacted more than ten regulations and policies since 1995 to clearly define the responsibilities associated with the different entities in the spent LIB recycling industry to deal with solid and hazardous wastes.^[^ ^9^ ^]^ These regulations cover nearly all aspects of LIBs, including LIBs’ production, collection, and recycling, and impose responsibilities for establishing a comprehensive management system for spent LIBs. China's regulations can provide references for other countries’ regulations to establish and manage spent LIBs. In 2019, more than 36% of spent LIBs in quantity were recovered in China^[^ ^9^ ^]^ and EVTank predicts that China's theoretical spent LIB recycling volume will reach 2.312 million tons in 2026.^[^ ^41^ ^]^ Although there are no government recycling centers for LIBs in China yet, more than 25 recycling companies are identified to be qualified for spent LIB recycling by now are specializing in LIB recycling, such as GEM Co., Ltd., Guangdong Brunp Recycling Technology Co., Ltd., and Hubei Fangyuan Environmental Protection Technology Co., Ltd.^[^ ^40b^ ^]^ However, policies can hardly be put in place due to the inadequate reward‐penalty system and regulatory tools.^[^ ^37c^ ^]^ As a result, spent LIBs often end up with small businesses, complicating the recycling process for formal recycling companies. China encourages battery manufacturers to implement multistage and multipurpose utilization of spent LIBs following the principle of cascade utilization before recycling under conditions that ensure safety and control. In addition, automakers must develop a collecting network for spent LIBs and use market mechanisms (buy‐back, trade‐in, and subsidies) to encourage EV users to trade in their spent LIBs. In 2019, China established a power battery recycling subsidy system in Shenzhen for the first time.^[^ ^41^, ^42^ ^]^ It is improving the subsidy policies from many perspectives with a dynamic coordination mechanism (based on price difference between oil and electricity, EV products performance, and number of charging facilities from year to year), adjusting the product structure and upgrading the products eligible for subsidies.^[^ ^9^ ^]^

#### 2.2.2 United States

In the US, so far, there has been little regulatory development in spent LIB recycling, with less than 1% of spent LIBs being recycled in the US.^[^ ^44^ ^]^ Market regulation is the focus for managing the LIB recycling industrial chain, with the government setting environmental protection standards and regulating them in a binding manner to help implement the recycling of spent LIBs. Legislation for spent LIB recycling in the US consists of federal, state, and local levels.^[^ ^1c^ ^]^ Federal, state, and local governments have jurisdiction over the disposal and recycling of LIBs.^[^ ^40a^ ^]^ Currently, there are only two federal laws related to LIB recycling: 1) Mercury‐Containing and Rechargeable Battery Management Act and 2) the Resource Conservation and Recovery Act. These two acts require battery companies and stores to collect spent batteries and establish a legal framework for disposing of LIBs as hazardous solid waste, respectively.^[^ ^40^, ^44^ ^]^ The federal government regulates battery manufacturers and spent battery recycling companies by issuing permits at the federal level. Moreover, while there are no state or local laws specific to LIB recycling, many states regulate the recycling of lead‐acid batteries. There are also several third‐party organizations for battery recycling in the US. One example is the Battery Council International (BCI). US public is generally aware of battery recycling, as many institutions popularize the recycling knowledge of spent LIBs. The US federal government funds research on how to recycle LIBs and supports the development of technologies to improve battery design and recycling, such as the US Department of Energy's ReCell Center.^[^ ^45^ ^]^ The US Congress also passed the Infrastructure Investment and Jobs Act to fund battery recycling programs in 2021.^[^ ^46^ ^]^

#### 2.2.3 European Union

The EU is one of the first regions to focus on battery recycling and taking action. In LIB recycling, the EU emphasizes EPR systems. Therefore, renewable energy EV manufacturers are actively taking action on recycling the spent LIBs. In Europe, various recycling policy frameworks exist as well.^[^ ^40a^ ^]^ However, there is currently no EU regulation on LIB recycling, while only a draft regulation to regulate the manufacturing, labeling, reusing, and recycling of batteries has been proposed. The GRS Batterien, jointly established by battery manufacturers and the Electrical and Electronic Industries Association, is Europe's largest LIB recycling organization and has been recycling industrial batteries since 2010.^[^ ^48^ ^]^ In the future, it will also include EV batteries in its recycling program and actively promote the recycling of LIBs. The EU has proposed taxation or subsidies for recycling and battery leasing (car manufacturers own the batteries and car owners pay for their use), but no relevant regulation has been implemented yet.^[^ ^49^ ^]^

#### 2.2.4 Impact of Regulations on LIB Recycling

For the development of LIB recycling regulations, due to the technical differences in battery design as well as utilization and the immaturity of technology in this area, the government should issue corresponding guidance documents and actively expand the regulation of EPR systems while allowing flexible implementation to facilitate establishing a large‐scale, efficient, and traceable LIB recycling management system. In this case, recycling regulations for lead‐acid batteries could serve as a wonderful template for LIB recycling regulations. In addition, strengthening publicity and education, establishing relevant organizations, and improving the overall public environmental literacy is vital.

On the contrary, the increased costs of regulations and standards can also be an obstacle to the commercialization of LIB recycling. Any LIB recycling policies should consider the potential hazards of LIB recycling and its mitigation for the environment and the communities in which they are recycled. A balance must be struck between cost efficiency, environmental sustainability, and worker health and safety to minimize the economic impact of LIB use and manufacturing while at the same time promoting technological advances.

### 2.3 Maximizing Profit and Protecting the Environment via LIB Recycling

The key to a successful recycling strategy is to maximize economic efficiency while minimizing environmental impact. Government regulations and other incentives (such as subsidies) are less critical if recycling is economically attractive.^[^ ^49^ ^]^ However, balancing cost efficiency, environmental sustainability, worker health and safety, and promoting technological advances while minimizing the economical impact of LIB use and production is not a small task.

#### 2.3.1 Local Recycling

While LIBs are hazardous materials, transportation costs account for a vital part of recycling costs.^[^ ^17^ ^]^ In the US, recyclers of LIBs are mainly located on the east and west coasts,^[^ ^17^ ^]^ while in China, recyclers of LIBs are mainly accumulated in the southeastern area.^[^ ^40b^ ^]^ Considering these centralized LIB recycling facilities, the LIBs discarded in the different areas must be transported long distances for recycling. Many LIBs (and other recyclable materials) are usually exported from the US and Europe to countries with lower labor costs (such as Asia). Plenty of wastes related to electricity, heat, toxic byproduct gases, pollution, human health hazards, etc., generated during collection, transportation, storage, and recycling of spent LIBs are undesirable. Once the volume of discarded LIBs reaches a significant level, more of the recycling sites should be established throughout the country for local recycling or preprocessing to achieve the best financial and logistical results.

#### 2.3.2 Increasing the Recovery Rate for Spent LIBs

China has many regulations, as discussed above; however, due to the lack of market standardization, price competition between small businesses and government‐approved companies for spent LIBs exists in a free‐market model. Many small businesses with mature industrial chains, low recycling costs, the cost of safety hazards, and environmental pollution can raise prices for buying spent LIBs. In this case, the high prices are the most significant competitive advantage.^[^ ^50^ ^]^ However, this method is not sustainable, and these small workshops are disrupting the LIB market's regular order by simply repairing and repackaging spent LIBs and returning them to the LIB market without relevant safety certifications. To a certain extent, it also leads to a failure of considerable, qualified LIB recycling manufacturers to receive spent LIBs for formal recycling under government supervision and legal restrictions due to the price. Without eliminating the disorder in the LIB recycling market, the recovery rate by a government‐certified and qualified LIB recycling company cannot be improved further, since the qualified LIB recycling companies cannot obtain enough LIBs for recycling to maintain the operation and profitability. Besides, establishing a battery‐tracking mechanism might be a viable solution to prevent spent LIBs from getting into the “black market.” In such a situation, each battery will be given an identification number, which will be uploaded into the tracking system through the utilization in the EoL value chain to facilitate the development of recycling.^[^ ^17^ ^]^

In addition, a “trade‐in” system would encourage more consumers to return their spent LIBs and ensure an adequate recovery number of spent LIBs. When consumers replace their old batteries with new ones, they can apply the old batteries toward the part of the price of the new batteries. EoL EV dismantlers should compensate consumers for recycling EVs with batteries; these dismantlers can sell the spent EV batteries to recyclers.

#### 2.3.3 Establishing a Practical Recycling Cost Analysis Model

**Figure 3:**
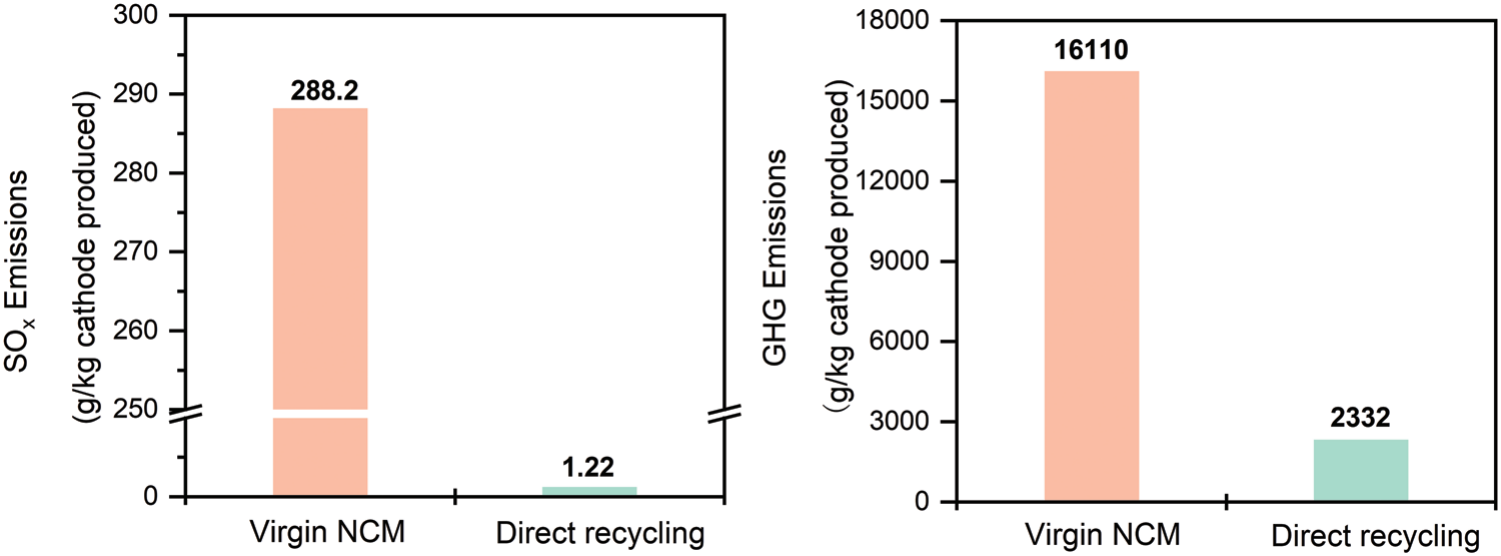
Reduction of SO*_x_* and greenhouse gas (GHG) emissions from recycling processes compared with primary cathode manufacturing.

Argonne National Laboratory announced the EverBatt model to the general public as a comprehensive recycling strategy evaluation tool, considering all steps from dismantling batteries from the vehicles to recycling. The EverBatt model can demonstrate the economic and environmental impacts of recycling methods, facilitating quantitative analysis for the LIB recycling process evaluation with economic and environmental impacts’ prediction.^[^ ^19b^ ^]^ Based on this model, with the advancement of LIB recycling, materials used in future EVs will likely be replaced with recycled materials, potentially reducing the total cost of a battery pack by up to 30%.^[^ ^15^ ^]^ In addition, battery disposal fees would be reduced thereby. Simultaneously, mitigating the adverse effects of new material extraction and disposal from spent LIBs is one of the most critical objectives of battery recycling. The LIB manufacturing process typically involves mining battery components (Co, Ni, and Cu) from the sulfide ore, which also generates significant amount of SO*_x_* and several other greenhouse gases (GHGs). Direct recycling, however, has a lower environmental impact, as shown in **Figure** **3**.

In conclusion, if the above challenges can be viewed as an opportunity to fully establish a sustainable recycling economy for LIBs, the economic and environmental benefits can be an essential driver for recycling LIBs.^[^ ^51^ ^]^

## 3 Conclusions and Outlooks

The resource shortage and the environmental impact of spent LIBs are gradually attracting attention as the demand for lithium batteries for EV increases and their lifespan ends. EoL batteries threaten the environment and human health, affecting the sustainable development of society. At the same time, it is also recognized that the spent LIBs are a vital resources for future battery materials. This paper summarizes the challenges and obstacles in developing LIB recycling, including room for technological improvement, policy gaps, the dilemma between securing economic benefits and environmental development of spent LIBs and presents opportunities based on existing challenges.

The following are recommendations that could facilitate the development of LIB materials’ recycling. Through the efforts of all parties involved, an automotive LIB recovery system can be realized even after many LIBs have been disposed of.

1) *On the Technical Side*: a) New recycling technologies can be developed to increase the value of recycled cathode materials: technologies such as upcycling, one way is high nickel NMC cathode formulations with the addition of virgin or recycled nickel to increase energy density and increase profits, and the other way is to convert polycrystalline material to monocrystalline material, resulting in better electrochemical performance. Another way is to utilize cathode surface modification or doping for enhanced cycling performance. b) Considering the end‐of‐life issues in cell designing and manufacturing, the cells and packs should be produced in a way that are easier to recycle. Standardizing the cell construction (cell module design and mechanisms) will reduce further recycling difficulties. Intelligent robots may replace the human workers to perform dangerous tasks, improving production efficiency and safety. c) The cooperation between the academic institutions or laboratories with industries can advance the direct recycling commercialization with more industrial‐level tests for the regenerated materials.
2) *Policy Considerations*: To speed the growth of LIB recycling, the government should establish funding priorities for R&D, pilot project funding, and market pull initiatives to set up a favorable investment climate for LIB collection and recycling. To begin with, it is recommended that the US federal funding for LIB collection be comparable to that for battery R&D research in order to grow the research budget and erase the disparity. Second, increased funding for pilot projects would fill data gaps in the industry recycling process, generate realistic data for investment planning, and give more verified data, allowing battery manufacturers to confirm the quality and compatibility of recycled materials. These will provide a more precise and dependable data set for the recycling process. More research data will assist analysts in becoming acquainted with closed‐loop recycling systems and identifying gaps and objectives for future study. Finally, favorable market pull policies can give early incentives and subsidies to support the development of LIB recycling technologies, scale up, and optimize the recycling value chain. Deposit reimbursement schemes might also incentivize consumers to recycle and properly dispose the spent LIBs. Creating a comprehensive battery recycling data monitoring platform and establishing effective regulations controlling the whole battery recycling process may support the improvement and growth of the battery collecting and recycling process.
3) *Economic and Ecological Considerations*: From the perspective of reducing economic and environmental costs, the economic efficiency of recycling LIBs should be improved by reducing production costs and increasing product value, recycling on‐site, reducing transportation costs, combining secondary use with recycling, mitigating the negative impacts of primary material extraction and LIB waste, minimizing the reduction of heavy metal element content, and selection of environmentally friendly binders and electrolyte systems are needed to reduce several environmental impacts.

We expect the EVs’ lithium battery recycling industry to gradually become more standardized and large‐scale over the next 5 years. As the residual value from battery recycling is increasingly exploited, consumers can use EVs at a lower cost. This benefit will further encourage battery and material manufacturers to enter the market, creating a virtuous cycle. In recent years, the scale of battery recycling is gradually snowballing. At the same time, there is a need to continuously compile and provide feedback on practical issues to improve policy standards and business models further.

## Conflict of Interest

The authors declare no conflict of interest.
